# Oral functions in adult persons with spinal muscular atrophy compared to a healthy control group: a prospective cross-sectional study with a multimodal approach

**DOI:** 10.1186/s13023-024-03405-5

**Published:** 2024-10-15

**Authors:** Teresa Kruse, Diana Leflerovà, Annette Cap, Sara Portegys, Brunhilde Wirth, Raoul Heller, Svenja Brakemeier, Tim Hagenacker, Bert Braumann, Gilbert Wunderlich

**Affiliations:** 1 Department of Orthodontics, Faculty of Medicine and University Hospital Cologne, University of Cologne, Kerpener Str. 32, 50931 Cologne, Germany; 2 Center for Rare Diseases Cologne, University of Cologne, Cologne, Germany; 3 Center for Molecular Genetics, University of Cologne, Cologne, Germany; 4 Present Address: Genetic Health Service NZ – Northern Hub, Auckland District Health Board, Auckland City Hospital, 90-102 Grafton Rd, Grafton, Auckland 1010 New Zealand; 5 Department of Neurology and Center for Translational Neuro- and Behavioral Sciences (C-TNBS), University Hospital Essen, Essen, Germany

**Keywords:** Spinal muscular atrophy, Bulbar neuromuscular function, Outcome measures, Bite force, Tongue pressure, Endurance

## Abstract

**Background:**

Oral function tests have been shown to reliably detect impaired bulbar function in adults with spinal muscular atrophy (SMA). Although not routinely recorded, it is known that persons with SMA are affected to varying degrees. Detecting differences in bite and tongue force, endurance, and maximum mouth opening has become particularly promising since the introduction of causal therapy for SMA. This study aimed to compare oral function among adult persons with SMA with different SMA types, walking abilities, and treatment status to a healthy control group.

**Methods:**

Data from oral function tests conducted on 58 persons with SMA and 45 healthy individuals were analyzed. Differences in oral function between SMA subgroups were pairwise tested and compared to the healthy control group using Wilcoxon rank sum tests.

**Results:**

In an overall comparison, three out of five oral function tests revealed lower values for the SMA group compared to the control group. Subgroup analyses indicated lower scores for most oral function tests in non-ambulatory, untreated patients with SMA type 2 compared to controls. Ambulatory, treated patients with SMA type 3 achieved strength and endurance values comparable to those of healthy individuals.

**Conclusions:**

The impairment of oral function varies across persons with SMA. Routine measurement of oral function is warranted to determine individual bulbar involvement stages. Further evaluation should be scheduled if indicators such as restricted maximum mouth opening arise.

*Trial registration* DRKS, DRKS00015842. Registered 30 July 2019, https://drks.de/register/de/trial/DRKS00015842/preview.

**Supplementary Information:**

The online version contains supplementary material available at 10.1186/s13023-024-03405-5.

## Background

Spinal muscular atrophy (SMA) is a severe neuromuscular disorder characterized by progressive degeneration of motor neurons in the spinal cord, with an incidence of 1 in 7.000 in Germany, based on newborn screening [1, 2]. SMA is caused by biallelic deletions and / or point mutations of the survival of motor neuron (*SMN*) 1 gene, leading to an SMN protein deficiency [3, 4]. The *SMN2* gene is a highly homologous copy of *SMN1* and is the major disease modifier [4, 5]. Its number of copies correlates inversely with disease severity [6–8].

The severity of muscular atrophy and weakness can vary considerably depending on age at onset of the disease, as well as treatment status. Historically, four different phenotypes have been defined with types 0–4 corresponding to decreasing motor function [9]. As gene-based therapies nusinersen (antisense oligonucleotide) and risdiplam (small molecule) correct the splicing of *SMN2* pre-mRNA, thereby increasing the number of functional SMN protein [10, 11]. Both significantly improve motor function and significantly alter the course of the disease [10, 12, 13].

In the natural disease course, progressive weakness and degeneration of bulbar muscles in persons with SMA inevitably lead to impaired muscle strength and decreased endurance in oral function. Postmortem studies of patients with SMA have shown involvement of the motor nuclei of the trigeminal, facial, and hypoglossal nerves, as well as the ambiguous nucleus, as correlates of limitations in bulbar function [14]. These limitations include impaired biting, chewing, and mouth opening, as well as dysarthrophonia, weak swallowing, and impaired airway protection [15]. While axial and proximal muscle groups are affected before bulbar muscles [16–18], minor oral symptoms, such as increased fatigability, may occur at earlier stages than currently assumed [19, 20]. In growing persons with SMA, impaired function of the jaw muscles alters craniofacial development and causes typical malocclusions that persist into adulthood. Typical are, among others, a retrognathia of the mandible with an increased overjet, an open bite due to excessive vertical development, rotations and displacement of the posterior teeth, and posterior crossbites [21, 22]. When a soft diet is required as a consequence, this, in turn, worsens malocclusions, further weakening maximum bite and tongue force [23, 24]. Deficits in swallowing and sucking as well as dysphagia are worsened by the weakness of the cervical muscles, increasing the risk of severe choking or aspiration pneumonia [25–27]. A lack of endurance of bite and tongue strength makes food intake even more difficult, increasing the risk of weight loss which may exacerbate the general condition of persons with SMA [28, 29]. Restricted mouth opening, limited lateral range of motion, and reduced protrusion of the mandible are known to be associated with atrophy and fatty infiltration of the lateral pterygoid muscle in persons with SMA [18, 25]. Morphological changes in the temporomandibular joints, including contractures, lead to a large proportion of rotation of the mandibular condyles at the expense of translation in the mouth opening movement [18, 30]. This progressive limitation of mouth opening, along with mandibular dysfunction and dysarthria, affects various aspects of speech, such as articulation, phonation, and resonance [31]. Complications can range from insufficient oral hygiene to potentially life-threatening situations, when for example an intubation is needed [29]. Consequently, bulbar dysfunction directly impacts patients’ quality of life and limits their daily activities [25, 32].

Previous research has demonstrated that combining measurements of maximum bite force, maximum tongue pressure, and maximum mouth opening covers a large part of bulbar muscle function and yields objective data across a continuous range [25, 33]. These oral function tests serve as appropriate instruments for regular clinical assessment as well as for outcome measures in clinical trials involving individuals with SMA [33].

The aim of this study was to compare persons with SMA and healthy individuals in terms of maximum bite force and endurance, maximum tongue pressure and endurance, as well as maximum mouth opening, while considering the heterogeneity in patients’ SMA type, walking ability, and treatment status.

## Material and methods

### Study design and study period

This prospective, cross-sectional multicenter study has been conducted from 2020 to 2022. Recruitment started in July 2020 and the first measurements were done in December 2020 until the end of this cross-sectional study period in December 2022.

### Participants and recruitment

A total of 58 adult individuals with genetically confirmed 5q-SMA and 45 adult healthy volunteers were included in this study. From the initial 59 persons with SMA recruited, one participant was excluded due to a maximum mouth opening of less than 8 mm, as it was not possible to place a bite force sensor. Individuals with SMA were recruited from the Departments of Neurology at the University Hospitals of Cologne and Essen, Germany, while healthy volunteers were recruited from the Dental School of the University of Cologne. All healthy volunteers underwent screening for craniomandibular dysfunction to ensure unbiased measurements. Sample size was determined by the number of eligible individuals willing to participate. The study was conducted in accordance with the Declaration of Helsinki and approved by the Ethics Committees of the Medical Faculties of the two sites (Reference Number Cologne: 19-1137; Reference Number Essen: 21-9851-BO). Every participant provided informed consent.

### Oral function measurements

Oral function tests were performed twice within one week on every person with SMA or healthy volunteer, during two separate visits. The tests were conducted at two sites and by one of three dentists who had received prior training. Administration procedures were standardized, and the order of evaluation was consistent for each measure analyzed. In the SMA group, repeated measurements were taken without the administration of nusinersen or other medical interventions in the interim or shortly prior to the tests. Patients receiving risdiplam therapy continued their medication as usual.

**Fig. 1:**
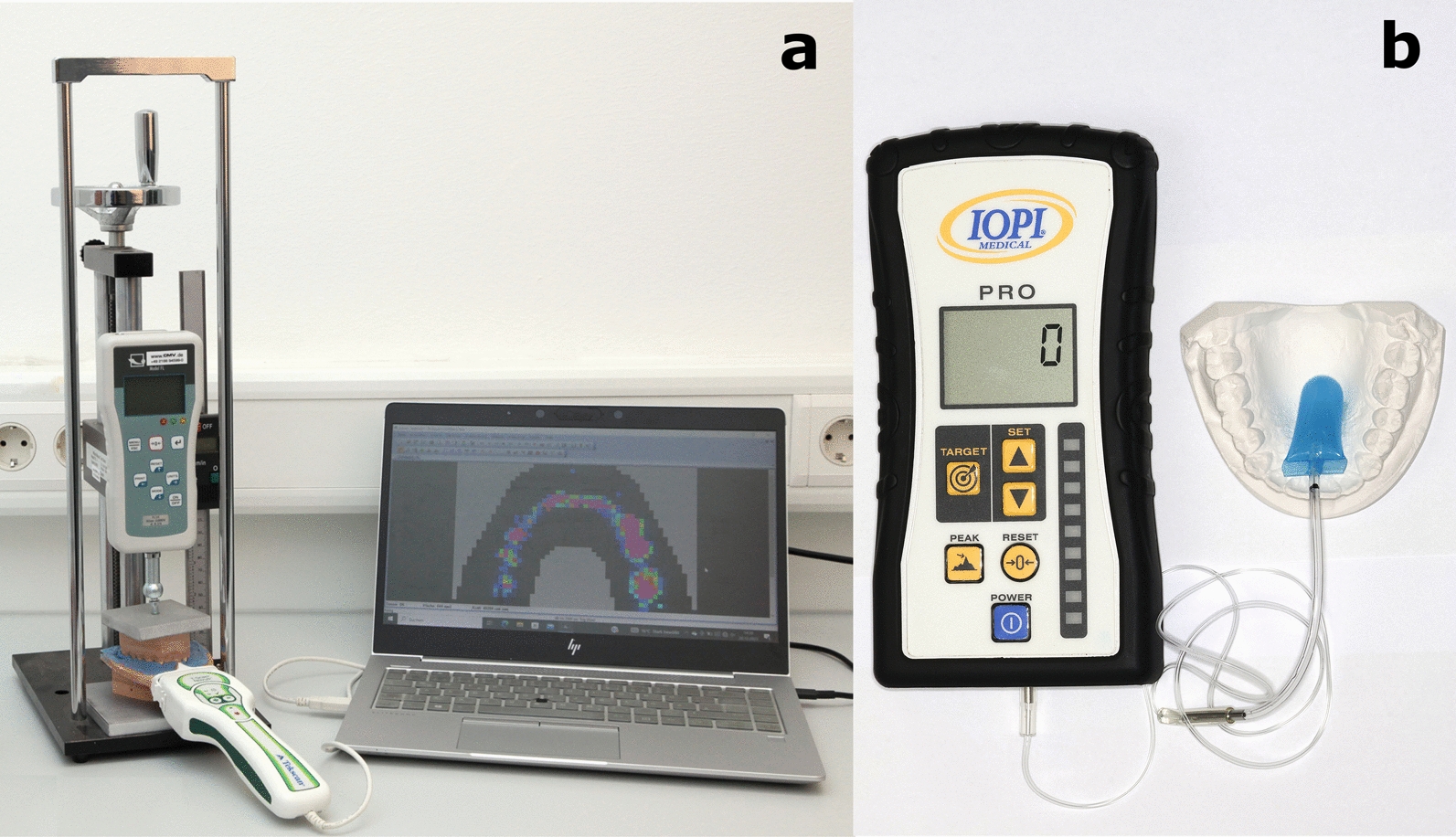
Measurement devices for bite force (**a**) and tongue pressure (**b**). **a** Individually adjusted piezoelectric T-Scan sensor foil with a T-Scan Handle and I-Scan software (Tekscan, Inc., South Boston, MA, USA). Shown here during the post-measurement calibration process with a participant's plaster casts clamped in place. **b** IOPI handheld device with air-filled bulb tongue array (IOPI Medical LLC, Carnation, WA, USA). The position of the bulb in a predefined midline position 10 mm posterior to the tip of the tongue and 10 mm anterior to the circumvallate papilla is displayed in relation to the dental arch

For measuring maximum bite force and bite force endurance, a piezoelectric T-Scan sensor was customized for each patient / volunteer using dental silicon to cover the entire dental arch [34]. Individualized sensors were calibrated with known loads, and the software I-Scan (Tekscan, Inc., South Boston, MA, USA) was used to record bite force values. Participants were instructed to bite three times with maximum force for three to four seconds with at least 30 s pauses between attempts to prevent muscle fatigue. The highest recorded score was used for analysis. Bite force endurance was evaluated by instructing patients / volunteers to maintain adduction at 60% of their previously established maximum bite force for as long as possible, with the duration measured until the value dropped below 30% of the predetermined maximum bite force level. Maximum tongue pressure and tongue pressure endurance were measured using a handheld device (IOPI Medical LLC, Carnation, WA, USA: Iowa Oral Performance Instrument) equipped with a single air-filled bulb tongue array [35]. The IOPI bulb was placed in a predefined midline position on the tongue blade: 10 mm posterior to the tip of the tongue and 10 mm anterior to the circumvallate papilla (Fig. 1). Participants were instructed to press the air-filled bulb against their palate with their tongue three times with maximum force for three to four seconds. Again, pauses of 30 s between each repetition were scheduled. The highest recorded score of the three attempts was used for analysis. Tongue pressure endurance was assessed correspondingly to bite force endurance, targeting a pressure level of 60%. Participants were able to visually monitor their 60% force level when measuring bite force endurance and tongue pressure endurance. Active maximum mouth opening was measured at the mesioincisal angle of the upper and lower front teeth using a ruler. An orthodontic examination of both groups was performed clinically and with the aid of plaster models. The evaluation included an assessment of sagittal dysgnathia and the relationship of the anterior teeth.

### Statistical analysis

Individual-specific data on five outcomes from two consecutive visits were analyzed. The mean across the first and second measurement was utilized for analysis. All outcome variables failed to withstand the Kolmogorov Smirnoff or Shapiro–Wilk test; no normal distribution could be confirmed. Differences in medians of oral function tests between the SMA group and the healthy control group were examined using Wilcoxon rank-sum tests for independent samples, assessing distributional differences. For subgroup analyses within the SMA group, patients were stratified by SMA type (SMA type 2 versus 3, with one patient with SMA type 1 and one with SMA type 4 excluded from this analysis of SMA type), by walking ability (ambulatory versus non-ambulatory), and by the treatment status (untreated versus treated). Wilcoxon rank-sum tests were again employed to compare medians of oral function tests, with pairwise comparisons made between SMA subgroups and the healthy control group. Additionally, ordinary least squares multiple regression was employed to assess whether patients’ SMA type or walking ability acted as confounders or moderators of observed differences between treated and untreated persons with SMA. Significance values were adjusted using Bonferroni correction for multiple testing, with statistical significance assumed at *p*-values < 0.05. All statistical analyses were conducted using SPSS 28.0.1.0 (IBM, SPSS statistics version 28.0.1.0, Chicago, IL, USA).

## Results

Sample characteristics are shown in Table 1. All persons with SMA receiving nusinersen therapy had initiated treatment at least six months prior to data collection. Seven persons with SMA received risdiplam treatment, either for a minimum of six months or as a switch from nusinersen to risdiplam therapy in direct temporal connection to the measurements. No patient had discontinuation of treatment. Individuals who were treatment-naïve at the time of the study, had not undergone any disease-modifying therapy (Table 1). The mean age among individuals who were untreated was 40.2 ± 13.5, compared to 37.1 ± 11.8 years among individuals receiving treatment.

**Table 1:** Characteristics of SMA study population

	Persons with SMA type 2		Persons with SMA type 3		Persons with SMA type 1		Persons with SMA type 4		Overall	
	Mean SD	N (%)	Mean SD	N (%)		N (%)		N (%)	Mean SD	N (%)
Age (years)	32.6 ± 7.8	21 (36.2)	41.0 ± 12.9	35 (60.3%)	19	1 (1.7%)	53	1 (1.7%)	37.8 ± 12.1	58 (100%)
Sex										
Male		9 (42.9%)		22 (62.9%)		0		1		32 (55.2%)
Female		12 (57.1%)		13 (37.1%)		1		0		26 (44.8%)
*SMN2* copies (number of)										
2		2 (9.5%)		0 (0%)		0		0		2 (3.4%)
3		16 (76.2%)		7 (20%)		1		0		24 (41.4%)
4		0 (0%)		28 (80%)		0		1		29 (50%)
n.a		3 (14.3%)		0 (0%)		0		0		3 (5.2%)
Walking ability										
Ambulatory		0 (0%)		15 (42.9%)		0		1		16 (27.6%)
Non-ambulatory		21 (100%)		20 (57.1%)		1		0		42 (72.4%)
Therapy										
Nusinersen		10 (47.6%)		28 (80%)		0		0		38 (65.6%)
Risdiplam		5 (23.8%)		2 (5.7%)		0		0		7 (12.1%)
Naïve		6 (28.6%)		5 (14.3%)		1		1		13 (22.4%)

*SD* standard deviations; *N* number; *n.a*. not available

Persons with SMA had moderate to severe malocclusions: More than half of the group (26 individuals) presented with retrognathia of the mandible, with some displaying an excessive overjet of over 10 mm (compared to the norm of 2 mm). Additionally, ten persons had an open bite of at least − 2 mm (compared to the norm of + 2 mm).

In the control group, out of 45 individuals, 13 were male and 32 were female, with a mean age of 23.9 ± 8.5 years (ranging from 17 to 57 years). Occlusion in the control group fell within the normal range [36–38]: 29 volunteers had orthognathia, ten had retrognathia of the mandible, six had prognathia of the mandible and/or retrognathia of the maxilla, and five individuals had an open bite of − 2 mm or more severe.

In an overall comparison of oral function between the healthy control group and the SMA group bite force endurance (*p* < 0.001) and maximum tongue pressure (*p* = 0.004) were lower in the SMA group compared to the control group. Additionally, persons with SMA had a smaller maximum mouth opening than those in the control group (*p* = 0.017). No differences were observed between the groups for maximum bite force and tongue pressure endurance (*p* > 0.05, Table 2).

**Table 2:** Comparisons between median values of healthy controls and persons with SMA

	Healthy control				Persons with SMA				*p*-value
	Median	IQR	Min	Max	Median	IQR	Min	Max	
Max. bite force (N)	560.43	223.95	289.90	1211.20	614.58	547.45	2.7	1394.05	0.549
Bite force endurance (sec)	65.25	18.00	29.5	168.50	43.48	19.80	10.45	96.85	< 0.001*
Max. tongue pressure (kPa)	53.00	18.50	33.50	72.00	44.75	29.50	9.00	88.00	0.004*
Tongue pressure endurance (sec)	29.50	20.50	15.00	146.50	31.23	20.88	11.50	93.50	0.756
Max. mouth opening (mm)	44.00	9.00	28.00	60.00	39.75	28.00	8.00	64.00	0.017*

*IQR* Interquartile range; *Min* minima; *Max* maxima; *p-value < 0.05

**Fig. 2:**
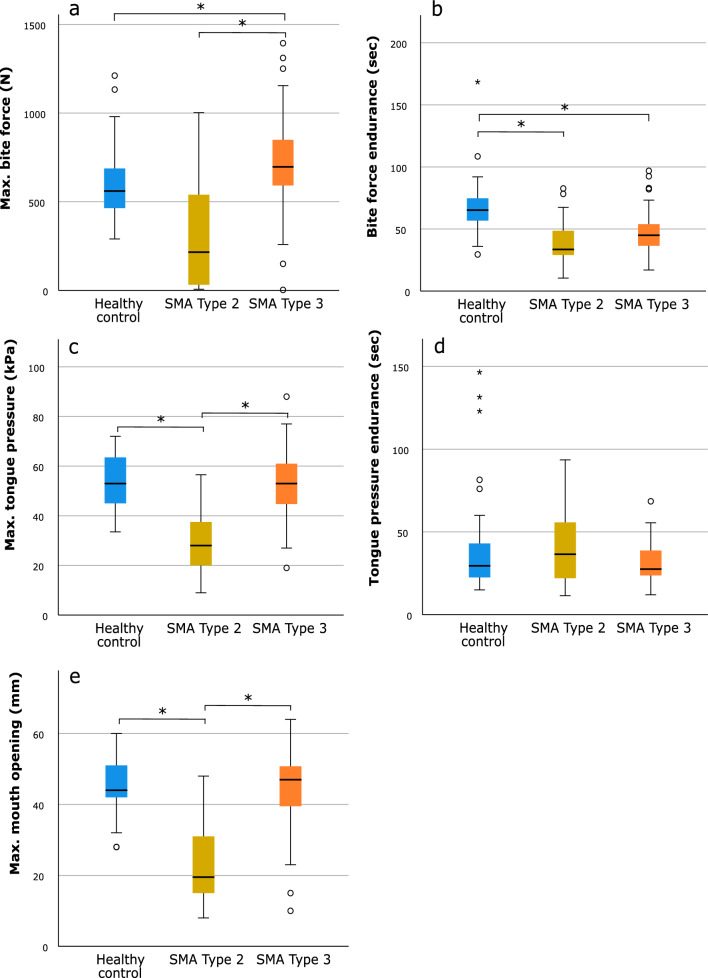
Pairwise comparisons among healthy controls and persons with SMA type 2 and 3. **a** Maximum bite force; **b** bite force endurance; **c** maximum tongue pressure; **d** tongue pressure endurance; **e** maximum mouth opening. Each box represents upper and lower quartiles with the median marked by a horizontal line in the box. Whiskers extend to the maximum value range, excluding outliers (denoted by circles, defined as lying more than 1.5 box-lengths outside the box) and extreme values (denoted by asterisks, defined as lying more than 3 box-lengths outside the box). *p-value < 0.05

Pairwise subgroup analyses revealed that persons with SMA type 3 had higher maximum bite force than those with SMA type 2 (*p* = 0.001) and than individuals in the control group (*p* = 0.007). No difference was found between SMA type 2 and the healthy control group for maximum bite force (Fig. 2a). Bite force endurance was higher in the control group compared to both SMA type 2 (*p* < 0.001) and SMA type 3 (*p* < 0.001), whereas no difference was observed between both SMA subgroups (Fig. 2b). Persons with SMA type 2 had lower maximum tongue pressure compared to both SMA type 3 (*p* < 0.001) and healthy controls (*p* < 0.001), and no difference was observed between SMA type 3 and healthy controls (Fig. 2c). No group differences were found for tongue pressure endurance (Fig. 2d). Persons with SMA type 2 had a smaller maximum mouth opening compared to both SMA type 3 (*p* < 0.001) and healthy controls (*p* < 0.001), whereas no difference was observed between SMA type 3 and healthy controls (Fig. 2e).

**Fig. 3:**
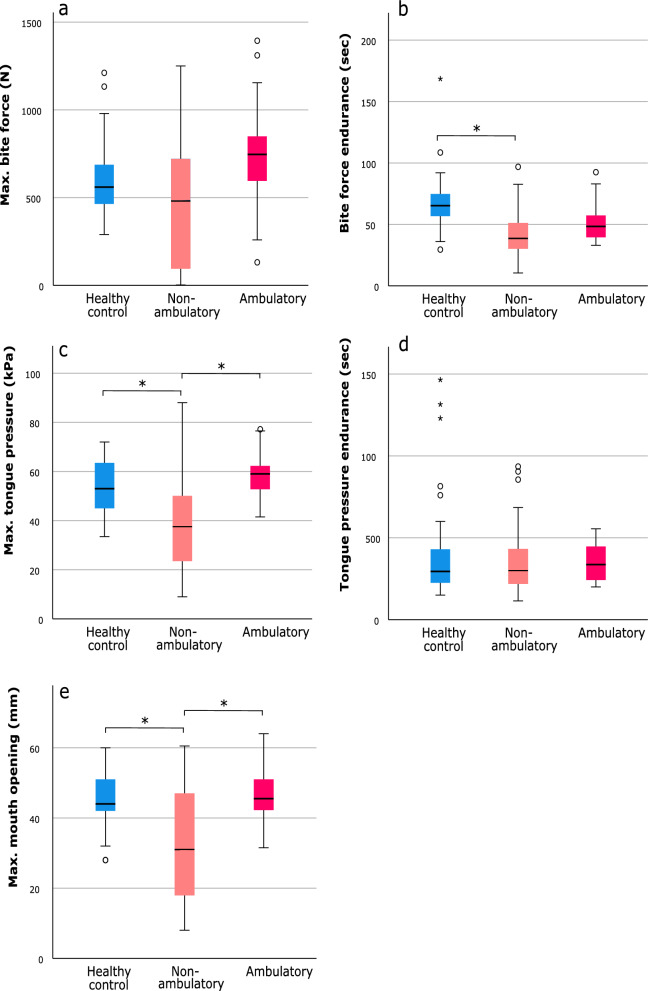
Pairwise comparisons among healthy controls and persons with SMA who were non-ambulatory or ambulatory. **a** Maximum bite force; **b** bite force endurance; **c** maximum tongue pressure; **d** tongue pressure endurance; **e** maximum mouth opening. Each box represents upper and lower quartiles with the median marked by a horizontal line in the box. Whiskers extend to the maximum value range, excluding outliers (denoted by circles, defined as lying more than 1.5 box-lengths outside the box) and extreme values (denoted by asterisks, defined as lying more than 3 box-lengths outside the box). *p-value < 0.05

In the classification according to walking ability, pairwise comparisons revealed that no group differences were observed for maximum bite force (Fig. 3a). Persons with SMA who were non-ambulatory had lower bite force endurance than those in the control group (*p* < 0.001). No other differences in bite force endurance were observed (Fig. 3b). Persons with SMA who were non-ambulatory had lower maximum tongue pressure compared to both healthy controls (*p* = 0.001) and persons with SMA who were ambulatory (*p* = 0.001), whereas no difference was observed between the latter and healthy controls (Fig. 3c). Pairwise comparisons for tongue pressure endurance revealed no differences between the groups (Fig. 3d). Persons with SMA who were non-ambulatory had a smaller maximum mouth opening compared to both ambulatory (*p* = 0.01) and healthy control groups (*p* = 0.003), and no difference was observed between the ambulatory and the control group (Fig. 3e).

**Fig. 4:**
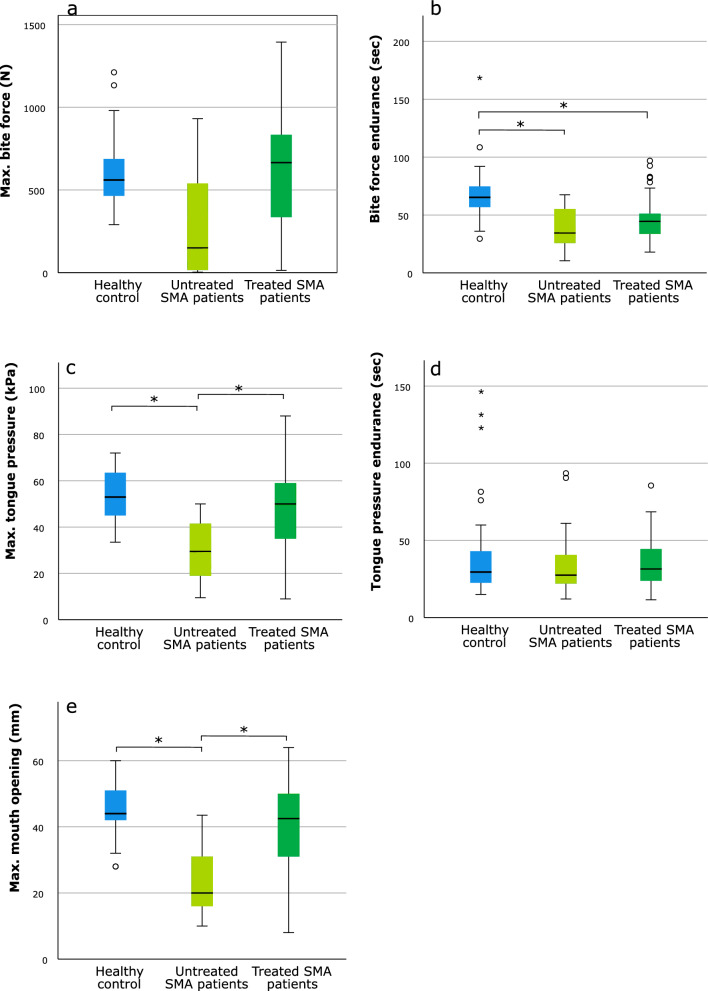
Pairwise comparisons among healthy controls and persons with SMA who were untreated or treated. **a** Maximum bite force; **b** bite force endurance; **c** maximum tongue pressure; **d** tongue pressure endurance; **e** maximum mouth opening. Each box represents upper and lower quartiles with the median marked by a horizontal line in the box. Whiskers extend to the maximum value range, excluding outliers (denoted by circles, defined as lying more than 1.5 box-lengths outside the box) and extreme values (denoted by asterisks, defined as lying more than 3 box-lengths outside the box). *p-value < 0.05

For maximum bite force, no differences were observed in the pairwise subgroup analyses between persons with SMA who were treated, those who were untreated, and the healthy control group (Fig. 4a). The untreated SMA subgroup had lower bite force endurance than the healthy controls (*p* = 0.017). Similarly, the subgroup of persons with SMA who received treatment had lower bite force endurance compared to controls (*p* < 0.001), whereas no difference was observed between both SMA subgroups (Fig. 4a). Persons with SMA who were untreated had lower maximum tongue pressure compared to both healthy controls (*p* < 0.001) and those of the treated SMA subgroup (*p* = 0.014), whereas no difference was observed between the latter and controls (Fig. 4c). For tongue pressure endurance no differences between the groups were observed (Fig. 4d). Pairwise comparisons of maximum mouth opening revealed that persons with SMA who were untreated had lower values than those who were treated (*p* = 0.0149) and than healthy controls (*p* = 0.003), whereas no difference was observed between controls and the treated SMA subgroup (Fig. 4e). All treatment-related results remained unchanged after controlling for patients’ SMA type and walking ability, with no evidence that these differences were specific to patients’ SMA type or walking ability (using multiple linear regression, see Additional file 1).

## Discussion

The results of this prospective, cross-sectional multicenter study confirm that oral function is diminished in persons with SMA compared to healthy controls. Significant differences were observed for bite force endurance, maximum tongue pressure and maximum mouth opening. Pairwise subgroup analyses revealed that higher values for several oral function parameters were measured in SMA type 3 versus type 2, ambulatory versus non-ambulatory, and in persons with SMA with versus without treatment.

While assessments of bite force, tongue pressure, and other measures of bulbar function have been conducted previously in persons with SMA and other neurodegenerative diseases [18, 39–41], this study provides novel data specifically on adult persons with SMA with and without treatment. Early assessments of oral function in treatment-naïve persons showed a reduction in maximum bite force by 19–50% compared to healthy controls [18, 41]. Bruggen et al. demonstrated evidence of mandibular dysfunction with bulbar involvement in patients with SMA type 2 and 3 [18]. Reduced tongue pressure has been associated with impaired bulbar and upper limb function in SMA, suggesting its utility as a biomarker [19]. Gwak et al. showed a correlation between restricted epiglottic retroflexion and lower tongue pressure in persons with SMA, emphasizing its importance for swallowing function [42]. These findings predate the availability of disease modifying therapies, which may explain why the extent of the reduction in maximum bite force observed in previous studies could not be replicated on our sample. Only 21.4% of patients had a limited mouth opening (≤ 30 mm), compared to 75% in the SMA sample studied by van Bruggen et al. [30].

Our results underscore the heterogeneity of oral function effort across persons with SMA, depending on characteristics such as SMA type, walking ability, and treatment status. Similar to findings by van Bruggen et al., our subgroup analyses showed lower values of maximum bite force (and maximum tongue pressure, as well as maximum mouth opening) in persons with SMA type 2 compared to type 3 [18]. This finding confirms previous reports of a higher incidence of bulbar dysfunction in SMA type 2 compared to type 3 [16]. The composition of our study samples shows an imbalance in favor of SMA type 3 (35 persons with type 3 versus 20 with type 2), which may explain the relatively high values observed in the overall analyses of maximum bite force and tongue pressure endurance. However, the ratio of patients who were ambulatory (16) to those who were non-ambulatory (42) could also influence these findings. Clinical observations suggest that dysphagia is rarely reported by patients who are ambulatory, and previous studies have identified correlations between walking ability and bulbar restriction [33]. Despite abnormalities in bulbar muscle structure, persons with SMA who are ambulatory can achieve similar masticatory performance as healthy controls [18, 32], a finding supported by our data. It is possible that less severely impaired patients can partially compensate for deficits in bulbar function, leading to better performance of certain oral function tests. Additionally, persons with SMA type 3 who are treated and ambulatory may not yet experience any restriction in oral / bulbar function, potentially due to the preserving effect of therapy. However, the reason for higher maximum bite force values in the SMA type 3 group compared to the control group remains unclear. Methodological inaccuracies in the measurement of maximum bite force, which are amplified by the typical orofacial anatomy in SMA, cannot be completely ruled out.

When controlling for differences concerning SMA type and walking ability, statistically significant differences remained between persons with SMA who were treated and those who were untreated, suggesting a therapeutic effect of disease modifying therapies on oral function in adult persons with SMA. While a multicenter observational study conducted in Germany found that persons with SMA who were ambulatory in particular benefited from therapy [43], our data suggest that they did not benefit differently from therapy compared to those who were non-ambulatory. To confirm this observation, changes in oral function over time should be monitored.

A limiting factor may be the lack of precise matching between the SMA and control group in terms of age and sex: 55.2% of the SMA sample were male compared to 29.5% in the control group; the mean age in the SMA group was 35.0 ± 14.5 versus 23.9 ± 8.5 in the control group. While the natural course of SMA generally involves progressive muscle weakness with increasing age [44], the onset of bulbar weakness or dysphagia may vary [27]. Previous studies provide conflicting evidence regarding age as an independent risk factor for limitations in oral function in SMA as well as in healthy individuals [29, 35, 45]. However, maximum bite force seem to remain constant in the period between the ages of 20 and 50 [46] which represents the majority of our participants. Robbins et al. found greater maximum tongue pressures in younger than in older individuals when the IOPI bulb was placed at the tongue blade site [35]. On the one hand, age-related reductions in muscle mass [47], changes in fiber density [48–51], fewer functional motor units [47] and central mechanisms may be involved, further exacerbated by age-related pathological processes [29, 45]. On the other hand, Robbins et al. noted that the placement of the bulb was also decisive. Methodological inaccuracies in the (sometimes difficult) placement of the bulb were therefore probably more decisive as potential confounder than age differences between the groups. Factors such as sex or malocclusion can affect maximum bite force, with differences in muscle strength between sex resulting in higher bite force level and maximum mouth opening in men [52–54] (albeit not necessarily in maximum tongue pressure [55–57]). However, differences in the number of teeth present may play a more significant role than age or sex in influencing maximum bite force [52, 58]. Due to a restricted mouth opening making oral hygiene and dental care difficult [30] patients with SMA often have poorer occlusion than healthy individuals. While the I-Scan / T-Scan system can compensate for differences in contact areas on the sensor, methodological compensation for anatomical alterations, including those of the musculature after tooth loss, is limited [59]. Moreover, craniofacial anomalies typical for SMA (such as retrognathia of the mandible and open bite) are associated with a reduced bite force [52, 60].

In order to preserve oral function, sensitive tests are crucial for early recognition of bulbar dysfunction, as interventions initiated late in the disease progression often yield diminished efficacy. Based on the findings of this study, we advocate for the implementation of a set of oral function tests capable of detecting oral dysfunction at an early stage, potentially even before patients themselves notice symptoms [19, 30]. While checking the maximum mouth opening can be realized by clinical providers and tongue pressure measurements are usually familiar to speech therapists, measurements of bite force should be left to specialized centers with affiliated dentists. In addition, the first signs of a functional oral impairment can also be seen during an annual check-up with a non-specialized general dentist and should be consistently reported to the treating neurologist. Upon detection, integrated oral rehabilitation for persons with SMA should include exercises targeting chewing, lip and tongue muscles [9].

## Conclusions

This comparison of oral function between persons with SMA and a healthy control group reinforces the existing evidence of diminished oral strength and endurance in SMA. First-time oral function assessments of adult persons with SMA on treatment show comparatively elevated values, suggesting a positive therapeutic impact on bulbar function in this specific group of patients. Notably, in the early clinical detection of oral / bulbar dysfunctions, a reduced maximum mouth opening compared to healthy reference values can be detected even without complex measuring devices by the clinical provider and should lead to further investigations.

## Supplementary Information

**Additional file 1.** Results from multiple linear regression models. Results from multiple linear regression models regressing patients' maximum tongue pressure and maximum mouth opening on their treatment status, controlling for their SMA type and ambulatory status (models 1 and 2), and on the interaction between treatment status and their SMA type and ambulatory status (models 3 and 4).

## Data Availability

The datasets used and/or analysed during the current study are available from the corresponding author on reasonable request in the figshare repository: https://figshare.com; 10.6084/m9.figshare.26324602 [61].

## Abbreviations

SMA: Spinal Muscular Atrophy
SMN: Survival Motor Neuron
SD: Standard Deviations
Min: Minima
Max: Maxima
IQR: Interquartile range
N: Number
n.a.: not available
