# Supplementary material for: Oral functions in adult persons with spinal muscular atrophy compared to a healthy control group: a prospective cross-sectional study with a multimodal approach
Source: Orphanet J Rare Dis. 2024 Oct 15;19:382. doi: 10.1186/s13023-024-03405-5 (PMC11481369; doi:10.1186/s13023-024-03405-5)
Supplement: Supplementary file 1 — Additional file 1. Results from multiple linear regression models. Results from multiple linear regression models regressing patients' maximum tongue pressure and maximum mouth opening on their treatment status, controlling for their SMA type and ambulatory status (models 1 and 2), and on the interaction between treatment status and their SMA type and ambulatory status (models 3 and 4). [file 13023_2024_3405_MOESM1_ESM.docx]

**Additional file 1**. Results from multiple linear regression models

| **Dep.var.:**  **Maximum tongue pressure** | M1 | | | |  | | M2 | | |  | M3 | | |  | M4 | | |
| --- | --- | --- | --- | --- | --- | --- | --- | --- | --- | --- | --- | --- | --- | --- | --- | --- | --- |
|  | coef | se | p-value |  | | coef | | se | p-value |  | coef | se | p-value |  | coef | se | p-value |
| Constant | 29.654* | 4.781 | < 0.001 |  | | 20.610* | | 4.082 | < 0.001 |  | 24.5* | 5.083 | < 0.001 |  | 28.542* | 4.459 | < 0.001 |
| Treated  (Yes / No) | 17.768* | 5.427 | 0.002 |  | | 11.671* | | 4.082 | 0.009 |  | 5.967 | 6.156 | 0.337 |  | 12.592* | 5.276 | 0.021 |
| SMA type 3/4 (Yes / No) |  |  |  |  | | 18.036* | | 4.111 | < 0.001 |  | 11.167 | 7.482 | 0.141 |  |  |  |  |
| Ambulatory (Yes / No) |  |  |  |  | | 9.349* | | 4.524 | 0.044 |  |  |  |  |  | 14.458 | 16.078 | 0.373 |
| Treated x SMA type 3/4 |  |  |  |  | |  | |  |  |  | 14.267 | 8.606 | 0.103 |  |  |  |  |
| Treated x Ambulatory |  |  |  |  | |  | |  |  |  |  |  |  |  | 4.408 | 16.804 | 0.794 |
|  |  |  |  |  | |  | |  |  |  |  |  |  |  |  |  |  |
| **Dep.var.:**  **Maximum mouth opening** | M1 | | | |  | | M2 | | |  | M3 | | |  | M4 | | |
|  | coef | se | p-value |  | | coef | | se | p-value |  | coef | se | p-value |  | coef | se | p-value |
| Constant | 23.692* | 3.895 | < 0.001 |  | | 15.186* | | 3.23 | < 0.001 |  | 18.929* | 3.892 | < 0.001 |  | 22.083* | 3.767 | < 0.001 |
| Treated  (Yes / No) | 15.091* | 4.422 | 0.001 |  | | 10.477* | | 3.415 | 0.003 |  | 4.988 | 4.714 | 0.295 |  | 12.692* | 4.457 | 0.006 |
| SMA type 3/4 (Yes / No) |  |  |  |  | | 17.564* | | 4.19 | < 0.001 |  | 10.321* | 5.729 | 0.077 |  |  |  |  |
| Ambulatory (Yes / No) |  |  |  |  | | 3.748 | | 4.726 | 0.3 |  |  |  |  |  | 20.917 | 13.581 | 0.129 |
| Treated * SMA type 3/4 |  |  |  |  | |  | |  |  |  | 11.979 | 6.59 | 0.075 |  |  |  |  |
| Treated * Ambulatory |  |  |  |  | |  | |  |  |  |  |  |  |  | -8.892 | 14.194 | 0.534 |
|  |  |  |  |  | |  | |  |  |  |  |  |  |  |  |  |  |

M1-M4: Models 1 – 4; Dep.var.: dependent variable; coef: coefficient estimate; se: standard error;

* p-value < 0.05
